# Simulation of the microwave five-band a perfect metamaterial absorber for the 5G communication‏

**DOI:** 10.1016/j.heliyon.2023.e19466

**Published:** 2023-08-26

**Authors:** Sarah Adnan Mohammed, Raed Ashraf Kamil Albadri, Khalid Saeed Lateef Al-Badri

**Affiliations:** Department of Physics, University of Samarra, Samarra, Iraq

**Keywords:** Penta-absorption bands, Metamaterial absorber, Microwave regime, 5G, SRR split-ring resonator

## Abstract

This study proposes a five-band perfect metamaterials absorber (MMA) for 5G communication in the K- and Ka-bands of the microwave range. The MMA design is based on a folded arms resonator (FAR) with a novel shape, forming the fundamental unit of the absorber. This absorber demonstrates a reasonably wide range of absorption capabilities for 5G communication in the K and Ka bands of the microwave region. The absorptivity of the MMA was examined for both normal and oblique incidence of waves in the frequency range of 20–26 GHz. According to a theoretical analysis, five absorption peaks at resonance frequencies of 20.38, 21.75, 23.1, 24.22 and 25.12 GHz exhibit absorption rates of 97.8%, 92.9%, 97.2%, 99.3% and 96.8%, respectively. The overall average absorption rate is 95.53%, taking into account the presence of two perfect absorption peaks. By adjusting the structural parameters, it is possible to influence the absorption peaks and resonant wavelengths. Additionally, the absorber demonstrates a high level of symmetry, resulting in insensitivity to TE mode polarisation angle and incident angle. The fractal resonators exhibited a capacitive effect at lower frequencies, while the SRRs demonstrated a capacitive effect at higher frequencies. This MMA design is expected to have practical applications in 5G communication technology.

## 1 Introduction

Metamaterials (MMs) are artificially engineered materials that consist of periodically repeated subwavelength-sized metallic or dielectric unit cells [[1], [2], [3], [4]]. These materials possess unique electromagnetic properties and have applications in various fields such as optical imaging [5], cloaking [6], antennas [7], sensors [3,[8], [9], [10], [11]], filters [12] and perfect absorbers [3,[9], [10], [11], [12], [13], [14], [15]].

Perfect absorption is an intriguing application of metamaterials (MMs), and extensive research has been conducted on perfect metamaterial absorbers (PMAs), showcasing narrow or wideband absorption characteristics depending on their specific applications. PMAs have found practical applications in wireless communication, emitters, sensors, photodetectors, photovoltaic solar cells and infrared camouflages [3,9,12,[15], [16], [17]]. The fundamental theory of Victor Georgievitch Veselago [18] first introduced the concept of metamaterials (MMs). These materials have proven to be highly useful in creating electronic circuits that can interact with electromagnetic waves of extremely small wavelengths [19]. One of the remarkable properties of MMs is their ability to demonstrate negative permittivities and/or permeabilities. [20,21], which justifies their alternative name, ‘double negative materials’ (DNG).

Researchers have reported various forms of PMAs suitable for operation in microwave, terahertz, visible and ultraviolet regions [[22], [23], [24]]. The desired absorption band is achieved by optimising the geometric parameters of the constituent meta-atoms, which are the building blocks of the metamaterial structure. PMAs typically consist of a top metasurface for incident wave penetration, a middle substrate to trap radiation and a bottom layer to block transmission. To increase the number of absorption bands, researchers have explored techniques involving multi-resonators in a super unit cell, multilayer structures, slot-loading, frequency selective surfaces and fractals. However, achieving multi-band absorption with a single metasurface layer remains a challenge due to drawbacks such as large size, fabrication complexities and high cost [[24], [25], [26], [27], [28], [29], [30]].

For example, in a previous study [31], an absorber based on square-shaped split-ring resonators (SRRs) was proposed specifically for Wi-Fi signal absorption. The design achieved two absorption peaks and exhibited polarisation insensitivity up to 90°, with angular stability of up to 40°. Another research work [32] discussed a triple-band absorber capable of absorbing frequencies at 8.5 GHz, 13.5 GHz and 17 GHz. However, the angular stability reported for this design was limited to 45°. In a different approach [33], a single-layer hybrid meta-structure was proposed for absorbing frequencies in the range of 2.6 GHz–18 GHz, using a dielectric material made of epoxy foam. Despite its wideband absorption capability, this design had a thickness of 3.2 mm and offered angular stability up to 30°. A quad-band absorber, designed with an anti-symmetric SRR, was reported in Ref. [34] for absorbing microwave frequencies at 4.1 GHz, 6.86 GHz, 11.3 GHz and 13.45 GHz. While the design showed excellent stability up to an incident angle of ***φ*** 60°, the angular stability was not specifically discussed. Additionally, in Ref. [35], a wideband metasurface absorber covering the 8 GHz–18 GHz band, achieving 80% absorption and polarisation insensitivity up to 45°, was proposed.

To overcome the limitations of single narrow-band absorption, there are typically two approaches employed to achieve multiple absorption bands. The first approach involves assembling multiple resonator structures with different geometric parameters in a coplanar configuration. Various studies have explored this approach, but it often results in larger unit cells, which contradicts the trend towards miniaturisation of absorbers [36]. The second approach involves vertically stacking multilayer subwavelength metallic structures to obtain multiband absorbers [[37], [38]]. However, this approach is constrained by fabrication processes, and the majority of reported designs are dual-band or triple-band absorbers, with little exploration of multi-band absorbers, especially those with five or more bands.

To introduce a new design strategy for multiband PMAs, we propose a novel design of a polarisation-insensitive five-band microwave PMA. The absorber exhibits five distinct absorption peaks at frequencies of 20.38, 21.75, 23.1, 24.22 and 25.12 GHz, with absorption rates of 97.8%, 92.9%, 97.2%, 99.3% and 96.8%, respectively. We also investigate the physical characteristics of the proposed PMA by analysing the electric and magnetic field distributions. Compared to previously reported multiband absorbers, our proposed absorber offers two main advantages. Firstly, its structure is simple, perfectly symmetrical and compact, making it suitable for microwave applications. Secondly, the absorption in the five-band PMA originates from the combination of dipolar resonances of the patterned metallic structure. This simple and compact PMA holds significant potential for various optoelectronic applications, including 5G, sensors, wireless communications and military systems.

## 2 The design and modelling of FAR-MMA

### 2.1 FAR-MMA based perfect metamaterial absorber presentation

**Fig. 1:**
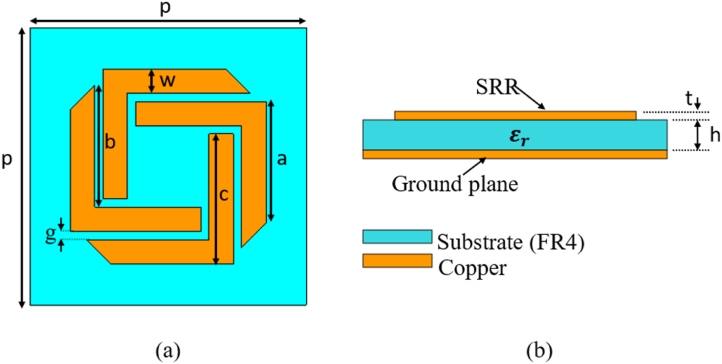
Depicts the unit cell design of the proposed metamaterial absorber (MMA). In (a), a top view of the Frequency-Selective Surface (FAR) is presented, while (b) illustrates the geometric layout of the MMA unit cell, highlighting various dimensions used in its construction.

The PMA is created by modifying the Frequency-Selective Surface (FSS) patch and adding a copper layer, which acts as the ground plane. These components are printed on opposite sides of the FR4 substrate. The design of the FAR patch is based on conventional SRRs with some modifications. Fig. 1(a) illustrates the physical structure of our designed PMA, while Fig. 1(b) and Table 1 showcase its periodic arrays on the plane. The PMA comprises three layers: the bottom layer (h3) and top layer (h1) are made of copper, and the intermediate layer (h2) is composed of an FR4 dielectric layer. The FR4 substrate has a thickness of 2 mm and a dielectric constant of εr = 4.3, with a tangent of loss tanδ = 0.002. The back metal layer, serving as a electromagnetic mirror, has an initial thickness of 35 μm with a copper conductivity of 5.8 × 10^7^ S/m.

**Table 1:** The optimal parametric values of the FAR-MMA.

Parameter	Value (mm)
a	7.5
b	7.5
c	8
g	0.5
h	2
p	17
t	0.035
w	1.5

To achieve 90% of absorption across multiple frequency bands, we simulated the unit cell structure using a frequency-domain solver in CST 2020 microwave studio (https://www.cst.com/). The simulation employed appropriate boundary conditions, including perfect magnetic and electric boundary conditions, and Floquet ports. By positioning the proposed design between two Floquet ports and exposing it to a uniform plan wave propagated along the z-axis under open-end boundary conditions, we extracted the electromagnetic responses. The unit cell condition was applied in both the x- and y-coordinates, with periodic boundary conditions along the x and y directions. The electromagnetic wave propagation was assumed to be in the negative direction along the z-axis. Moreover, the electric field (E) and magnetic field (H) were oriented in the positive direction along the y-axis and x-axis, respectively.

### 2.2 Microwave absorption in metamaterial resonator

The interaction between electromagnetic (EM) waves and the MMA plays a significant role in determining its physical properties. This phenomenon can be categorised into two cases: normal incidence and oblique incidence. In microwave structures, the power gain is enhanced through radiation absorption. Conversely, significant transmission and/or reflection can lead to reduced absorption, as described by Equation (1) [3,7,39].

(1) $$A\left(\omega \right)=1-R\left(\omega \right)-T\left(\omega \right)=1-{\left|S_{11}\left(\omega \right)\right|}^{2}-{\left|S_{21}\left(\omega \right)\right|}^{2}$$

This equation establishes a connection between the absorption coefficient (A(ω)), the reflection coefficient (R(ω)), and the transmission coefficient (T(ω)). Therefore, achieving significant absorption coefficients relies on minimizing the values of both R and T.

The ground plane of the FAR is made of copper, a highly conductive material with a conductivity (σ) of 5.8 × 10^7^ (s/m) and a resistivity (ρ) of 1.72 × 10^−8^ (Ω-meter). The ground plane's small skin depth (δ) compared to its thickness (35 μm) results in a significant weakening of transmission within the ground plane (S_21_ = 0).

The design of the non-printed areas is customized to attenuate or absorb specific wavelengths or a range of wavelengths. Larger patterns are effective in absorbing longer wavelengths and lower frequencies, while smaller patterns can absorb shorter wavelengths and higher frequencies. As a result, absorbent metamaterials offer advantages like reduced thickness and mass. These absorbers can be produced using standard materials in the printed circuit industry for microwave frequency bands, meeting production requirements [[17], [18], [19], [20]].

**Fig. 2:**
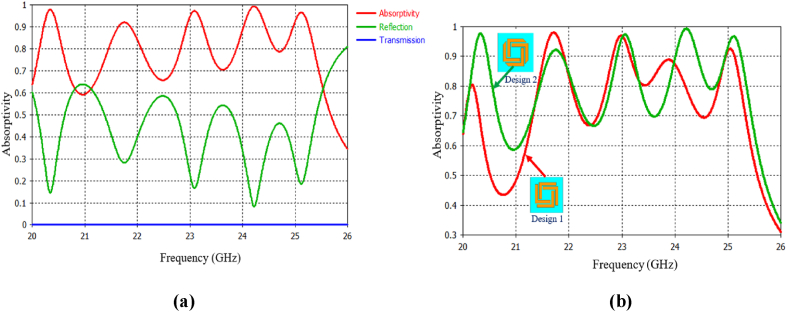
(a) Spectral response of the FAR unit cell vs. frequency. (b) Absorption spectra of Design 1.

The combined structure depicted in Fig. 2 demonstrates the individual effects of all the resonators. It exhibits a five-band response with resonances occurring at frequencies of 20.38, 21.75, 23.1, 24.22 and 25.12 GHz, exhibiting absorption rates of 97.8%, 92.9%, 97.2%, 99.3% and 96.8%, respectively.

On the other hand, as the impedance of the unit cell approaches the characteristic impedance (Z_o_) of free space (377 Ω) from which the EM wave is incident, the reflection coefficient (S_11_(ω)) in Equation (1) approaches zero at the desired absorption frequency. This is achieved by the tight coupling between the FAR arranged symmetrically in the proposed absorber. The reflection coefficient becomes zero (S_11_(ω) = 0) at multiple frequency bands when the impedance (Z(ω)) matches the characteristic impedance (Z_o_) of free space, allowing for absorption of the desired frequencies.

To examine the impact of design variations on absorption spectra, two distinct designs were studied: Design 1 and Design 2 (proposed design). As shown in Fig. 2(b), Design 1 displayed a decrease in absorption magnitude and a shift in resonance frequency, primarily caused by modifications in the resonator shape. Consequently, Design 1 did not demonstrate favorable absorption characteristics and would not be suitable for practical applications. In contrast, the proposed structure exhibited perfect absorption across all five frequency bands, making it highly suitable for practical use.

## 3 Results and discussion

### 3.1 Relative permeability and relative permittivity

Fig. 2 displays the absorption characteristics of the intended absorber, showcasing distinct resonance frequencies and absorption coefficients for each model. The determination of permittivity and permeability involves the Nicolson-Ross-Weir technique implemented in MATLAB software. To investigate the metamaterial (MTM) property of the proposed MMA, we examine the real and imaginary effective permittivity (ε) responses and permeability (μ) response of the absorber.

**Fig. 3:**
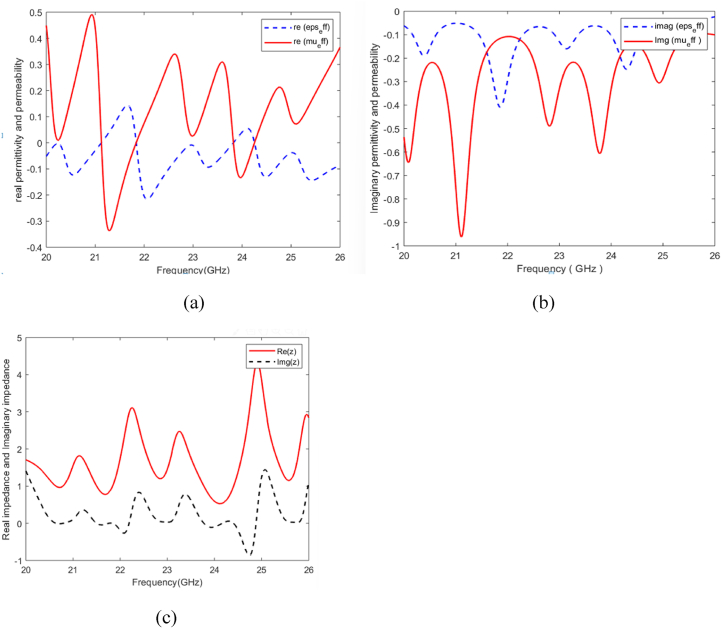
Illustrates the real and imaginary components of (a) Relative Permeability, (b) Relative Permittivity, and (c) Real and imaginary impedance..

Fig. 3(a) and (b) illustrate that the relative permeability exhibits negativity at specific frequencies: 20.38 GHz, 21.75 GHz, 23.1 GHz, 24.22 GHz, and 25.12 GHz. Similarly, Fig. 3 demonstrates the negativity of the relative permittivity at the same frequencies. As a result, the refractive index also shows negativity at 20.38 GHz, 21.75 GHz, 23.1 GHz, 24.22 GHz, and 25.12 GHz. These findings lead to the conclusion that the proposed MMA possesses double-negative properties at the absorption frequencies.

The effective permittivity and permeability responses of the proposed MTM absorber, depicted in Fig. 3(a) and (b), are plotted using the equations mentioned in Ref. [40], where the effective permeability and permittivity are represented by Eqns. (2), (3) respectively.

(2) μ=nz

(3) $$\varepsilon =\frac{n}{z}$$

The impedance (z) and refractive index (n) terms in Eqns. (2), (3) are obtained from Eqns. (4), (5), respectively.

(4) $$z=\pm \sqrt{\frac{{\left(1+S_{11}\right)}^{2}-S_{21}^{2}}{{\left(1-S_{11}\right)}^{2}-S_{21}^{2}}}$$

(5) $$n=\frac{1}{k_{0}d}\left[{\left[\ln\left(e^{ink_{0}d}\right)\right]}^{\prime \prime }-i{\left[\ln\left(e^{ink_{0}d}\right)\right]}^{\prime }\right]$$

Where $S_{21}$ and $S_{11}$ in Eqn. (4) denote the (R(ω)) and (T(ω)) of the metamaterials-based absorber. In Eqn. (5) the $e^{ink_{0}d}$ term is denoted by $e^{ink_{0}d}=\frac{S_{21}}{1-S_{11}\frac{z-1}{z+1}}$ , where ${\left[\ln\left(e^{ink_{0}d}\right)\right]}^{\prime }$ and ${\left[\ln\left(e^{ink_{0}d}\right)\right]}^{\prime \prime }$ represents the real and imaginary part of the $e^{ink_{0}d}=\frac{S_{21}}{1-S_{11}\frac{z-1}{z+1}}$ respectively. In this context, *k*_0_ represents the wavenumber, and *d* represents the maximum length of the unit cell. The entire MTM perfect absorber is treated as a single unit cell.

Moreover, the minimal reflections from the surface of the proposed structure indicate excellent impedance matching between the absorber surface impedance and free space impedance. This implies that at resonance frequencies, the real component of normalized impedance approaches unity, while the imaginary component reduces to zero. Through the optimization of the structural parameters of the metamaterial resonator to control the permittivity and permeability, the impedance of the two-absorption band aligns with the free space impedance (refer to Fig. 3(c)).

### 3.2 TE polarizations

The main determinant of the absorption efficiency in a metamaterial absorber (MMA) is its interaction with incident electromagnetic waves at the top, which is smaller than the wavelength. Typically, resonant patterns in MMAs generate only a single absorption band. However, the proposed structure possesses rotational symmetry, leading to consistent absorption results for both TE polarizations under normal incidence. Fig. 2 shows the absorption spectrum of the proposed Frequency-Selective Surface (FAR) under TE, displaying five absorption peaks when waves are incident normally with varying polarisation angles. Despite its ultrathin thickness of 0.075 λ₀ (where λ₀ represents the wavelength of the lowest frequency [32]), the MMA achieves unity absorption at different peaks. Moreover, due to its symmetrical geometry, the absorber exhibits polarisation insensitivity under normal incidence. This well-designed absorber can effectively serve as an electromagnetic (EM) detector for GHz frequencies.

**Fig. 4:**
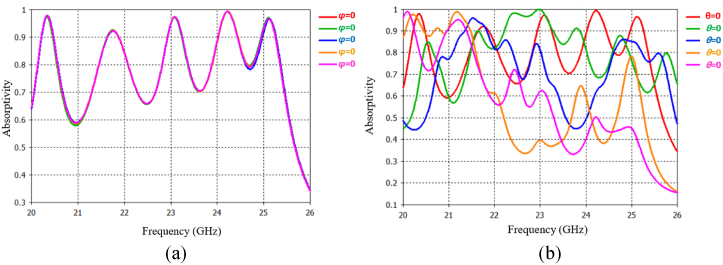
(a) Absorptivity of the proposed FAR at different φ angles. (b) Absorptivity of the proposed FAR at different θ angles.

Consequently, Fig. 4 depict the absorption properties of the proposed MMA at different incidence angles. As shown in Fig. 4(a), the five resonance peaks remain unchanged as the incident angle (φ) varies from 0° to 60°. Furthermore, even at incidence angles up to 60°, the absorption remains consistently above 90%. Similarly, Fig. 4(b) demonstrate that the five absorption peaks maintain stability, with their absorption coefficients remaining above 50% even at a 45-degree incident angle. Additionally, it was found that certain oblique incidences result in additional peaks due to the possibility of a similar decrease in electromagnetic radiation and material absorption [30].

**Fig. 5:**
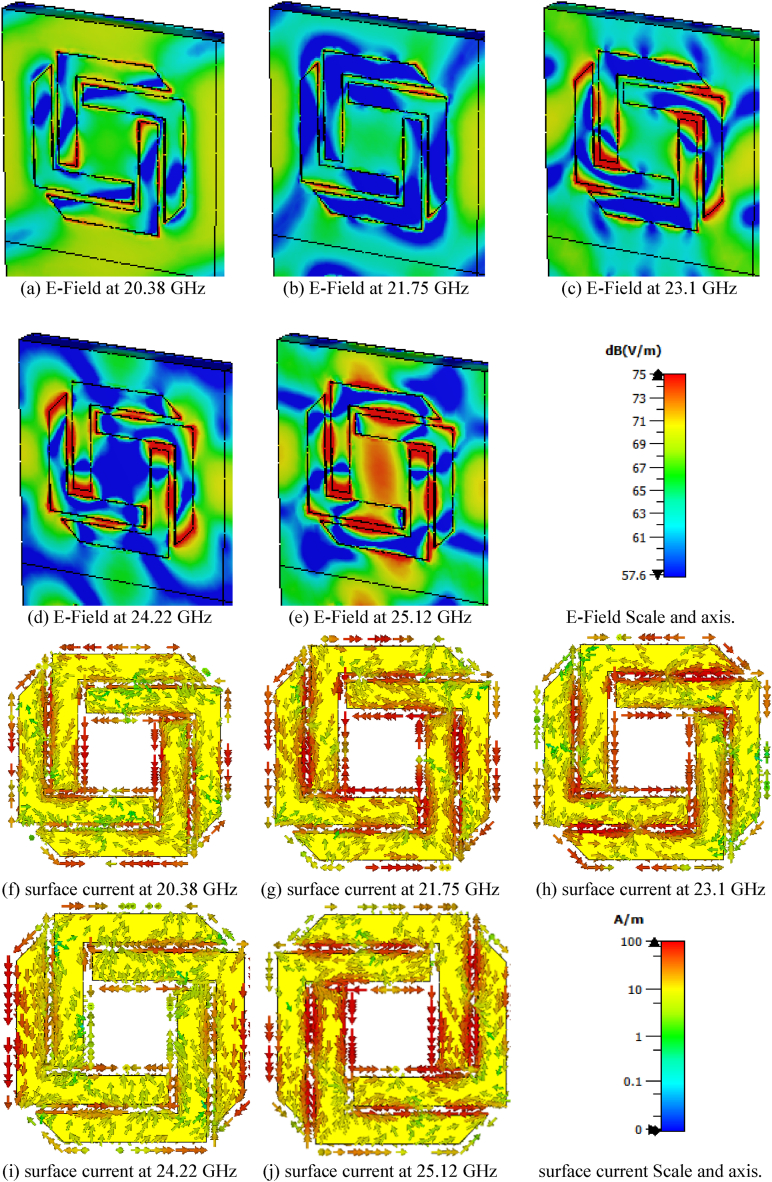
The electric field distribution at (a) 20.38 GHz, (b) 21.75 GHz, (c) 23.1 GHz, (d) 24.22 GHz and (e) 25.12 GHz, and surface current for (f) 20.38 GHz, (g) 21.75 GHz, (h) 23.1 GHz, (i) 24.22 GHz and (j) 25.12 GHz.

Fig. 5(a)-(j) depict the electric field distribution and surface current for each absorption peak at 20.38 GHz, 21.75 GHz, 23.1 GHz, 24.22 GHz, and 25.12 GHz, respectively. At 20.38 GHz, 21.75 GHz, and 23.1 GHz, the z-component absolute of the Ez-field distributions (i.e., z-component) shows maximum electric field concentration along the end point of FAR arms (Fig. 5(a)–(e)), indicating the occurrence of electrical resonance. Simultaneously, the surface current distribution at the same frequencies exhibits opposite orientations, signifying the occurrence of magnetic resonance. Thus, it is evident that both electric and magnetic resonance occur at 20.38 GHz, 21.75 GHz, and 23.1 GHz, resulting in perfect absorption.

At 24.22 GHz and 25.12 GHz, electric and surface resonance occur simultaneously at the center and along the four inside arms (Fig. 5(f)–(j)). The surface current in the top forms a circulating current that induces magnetic excitation.

### 3.3 Unit cell design parameters

In this section, a comprehensive parametric study is presented to gain a thorough understanding of the behavior of the proposed FAR. During the design process, the FAR is affected by variations in the resonator copper width and the substrate thickness.

#### 3.3.1 Variation of copper width

**Fig. 6:**
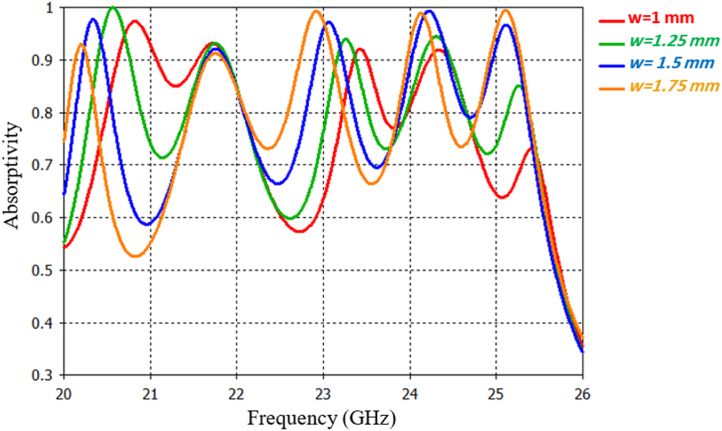
Absorption for various arm widths.

Specifically, in Section 3.4.1, the variation of the copper width is analyzed as it plays a crucial role in the behavior of metamaterial Split Ring Resonators (SRRs). The FAR consists of a total of eight arms (w), and the reflection coefficient (S₁₁) of the MMA is analyzed for four different width values (w = 1 mm, 1.25 mm, 1.5 mm, and 1.75 mm). Fig. 6 illustrates the reflection characteristics for various w values, revealing that resonance frequencies are directly proportional to the w values. Increasing the w values leads to a decrease in resonance frequencies, and vice versa. Reducing the w width decreases the capacitance of the resonator, resulting in increased resonances in the reflection spectrum. Among the evaluated w values, a width of 1.5 mm exhibits superior performance and is thus chosen for the design of the proposed structure.

#### 3.3.2 Substrate thickness effect

**Fig. 7:**
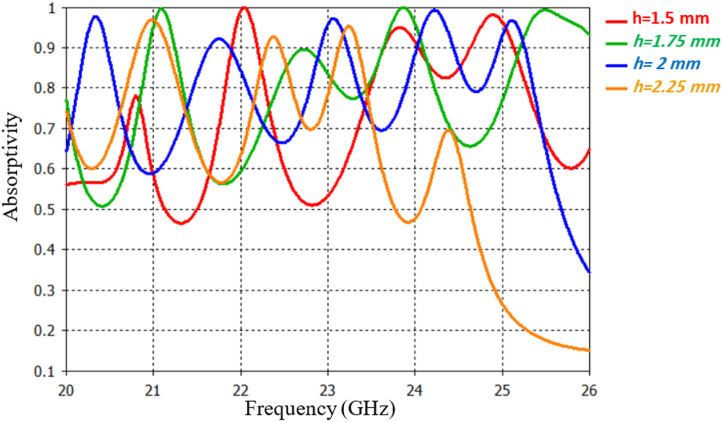
Absorption for various substrate thicknesses.

The study also investigates the impact of different thicknesses of the FR4 substrate (denoted as h) on absorption performance. Four different substrate thickness values are considered: h = 1.5 mm, 1.75 mm, 2 mm and 2.25 mm. As depicted in Fig. 7, the absorption characteristics, including resonance frequencies and absorption coefficients, vary depending on the substrate thickness value. In contrast to the previous case, an increase in substrate thickness leads to a decrease in resonances and vice versa. Among the examined thickness values, a thickness of 2 mm yields the highest absorption rates with the desired adaptation. Therefore, this value is chosen for the design of the FAR, which forms the MMA.

### 3.4 Equivalent circuit model

**Fig. 8:**
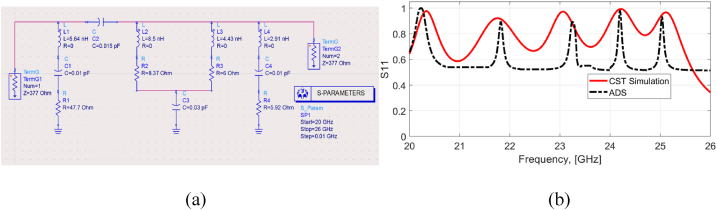
(a) The equivalent circuit of the unit cell, (b) S11 curve from CST and ADS.

The circuit model of the proposed absorber is shown in Fig. 8. The values of inductance (L) and capacitance (C) are determined based on Fig. 2, taking into account the resonance frequencies and the length of L and C obtained from the surface current distribution in Fig. 5. The resistance (R) values for each L-C-R circuit, corresponding to each resonance frequency, were adjusted using the ADS (Advanced Design System) software 2021, a circuit simulator based in Carlsbad, CA, USA, to match the corresponding S11 and S21 parameters obtained from CST simulation. Fig. 8(b) demonstrates that the S11 parameters obtained from the ADS circuit and CST simulation reasonably match at the resonance frequencies, indicating good agreement for the reflection coefficient.

Finally, a comparative study of the proposed MMA and newly published MMA is presented in Table 2 to highlight the novelty of the studies in this suggested work.

**Table 2:** Performance comparison of the proposed MMA with related works.

Ref.	Number of absorption bands	Absorption peak resonance (GHz)	Frequency band	Structure complexity	Other factors
[9]	1	3.374	S	Simple structure	Single narrow band based on hole ring
[41]	2	6.45, 14.89	C and Ku	Complex structure	Double narrow bands based on Tuning fork shape
[42]	3	4.1, 6.1, 10.1	S, C and X	Complex structure	Narrow bands based on three anchors
[43]	3	4.2, 5.6, 7.7	C	Simple structure	Three broad bands and polarisation sensitive.
[44]	4	7.09, 9.25, 10.42, 12.94	C, X and Ku	Complex design	Four bands based on Dual-T Circular Shape
This work	5	20.38, 21.75, 23.1, 24.22, 25.12	K and Ka	Simple structure	Five broad bands based on folded arms resonator

From this comparative analysis, we can draw the conclusion that the proposed MMA is compact in size and features a simple design approach, using a single metamaterial resonator cell. Additionally, the MMA can serve as both an absorber and a sensor, making it suitable for various applications in the biomedical field.

## 4 Conclusions

In conclusion, this article presents an effective MMA design that optimises absorption rates in the desired K and Ka frequency bands. The MMA uses a unique FAR configuration, enabling rotational symmetry and near-unity absorption. It exhibits polarisation angle insensitivity and angle insensitivity to incident waves. The article provides a comprehensive analysis of constitutive parameters and electromagnetic field variations. Based on simulation results, the presented design offers a compact and ultrathin absorber, achieving five absorption bands with nearly perfect absorption peaks. Notably, the design has a compact and ultrathin profile, with a thickness of 0.075 times the free space wavelength at the first absorption peak. This MMA design holds great potential for applications in 5G, sensors, wireless communications and military systems.

## Author contribution statement

Sarah Adnan Mohammed: Conceived and designed the experiments; Contributed reagents, materials, analysis tools or data; Wrote the paper.

Raed Ashraf Kamil Albadri: Conceived and designed the experiments; Contributed reagents, materials, analysis tools or data.

Khalid Saeed Lateef Al-Badri: Performed the experiments; Analyzed and interpreted the data.

## Data availability statement

No data was used for the research described in the article.

## Additional information

No additional information is available for this paper.

## Declaration of competing interest

The authors declare that they have no known competing financial interests or personal relationships that could have appeared to influence the work reported in this paper.
